# Quaternary structure of patient-homogenate amplified α-synuclein fibrils modulates seeding of endogenous α-synuclein

**DOI:** 10.1038/s42003-022-03948-y

**Published:** 2022-09-30

**Authors:** Benedikt Frieg, James A. Geraets, Timo Strohäker, Christian Dienemann, Panagiota Mavroeidi, Byung Chul Jung, Woojin S. Kim, Seung-Jae Lee, Maria Xilouri, Markus Zweckstetter, Gunnar F. Schröder

**Affiliations:** 1 Institute of Biological Information Processing (IBI-7: Structural Biochemistry), Forschungszentrum Jülich GmbH, Wilhelm-Johnen-Straße, 52425 Jülich, Germany; 2 German Center for Neurodegenerative Diseases (DZNE), Von-Siebold-Str. 3a, 37075 Göttingen, Germany; 3 Department of Molecular Biology, Max Planck Institute for Multidisciplinary Sciences, Am Faßberg 11, 37077 Göttingen, Germany; 4 Center of Clinical, Experimental Surgery, & Translational Research, Biomedical Research Foundation of the Academy of Athens (BRFAA) 4, Soranou Efesiou Street, Athens, 11527 Greece; 5 Department of Biomedical Sciences, Neuroscience Research Institute, College of Medicine, Seoul National University, Seoul, 03080 Korea; 6 Brain and Mind Centre and School of Medical Sciences, Faculty of Medicine and Health, The University of Sydney, Sydney, NSW Australia; 7 School of Medical Sciences, University of New South Wales & Neuroscience Research Australia, Randwick, NSW 2031 Australia; 8 Department for NMR-based Structural Biology, Max Planck Institute for Multidisciplinary Sciences, Am Faßberg 11, 37077 Göttingen, Germany; 9 Physics Department, Heinrich Heine University Düsseldorf, Düsseldorf, Germany; 10 Present Address: Nutritional Sciences and Toxicology Department, University of California Berkeley, Berkeley, CA 94720 USA

**Keywords:** Cryoelectron microscopy, Mechanisms of disease, Prions

## Abstract

Parkinson’s disease (PD) and Multiple System Atrophy (MSA) are progressive and unremitting neurological diseases that are neuropathologically characterized by α-synuclein inclusions. Increasing evidence supports the aggregation of α-synuclein in specific brain areas early in the disease course, followed by the spreading of α-synuclein pathology to multiple brain regions. However, little is known about how the structure of α-synuclein fibrils influence its ability to seed endogenous α-synuclein in recipient cells. Here, we aggregated α-synuclein by seeding with homogenates of PD- and MSA-confirmed brain tissue, determined the resulting α-synuclein fibril structures by cryo-electron microscopy, and characterized their seeding potential in mouse primary oligodendroglial cultures. The combined analysis shows that the two patient material-amplified α-synuclein fibrils share a similar protofilament fold but differ in their inter-protofilament interface and their ability to recruit endogenous α-synuclein. Our study indicates that the quaternary structure of α-synuclein fibrils modulates the seeding of α-synuclein pathology inside recipient cells. It thus provides an important advance in the quest to understand the connection between the structure of α-synuclein fibrils, cellular seeding/spreading, and ultimately the clinical manifestations of different synucleinopathies.

## Abstract (web summary)

Quaternary structure of α-synuclein fibrils characterized from human patients with Parkinson’s disease and Multiple System Atrophy modulates seeding in mouse primary oligodendroglial cultures.

## Introduction

α-synucleinopathies are neurodegenerative diseases in which the presynaptic protein α-synuclein (αSyn) misfolds and abnormally aggregates into fibrils. In the case of Parkinson’s disease (PD) and dementia with Lewy bodies, these αSyn fibrils are abundant in Lewy bodies within neurons or Lewy neurites^1^. For multiple system atrophy (MSA), αSyn fibrils are predominantly found in inclusions within oligodendrocytes, with limited pathology in neurons^2^. Remarkably, αSyn directly transmits and self-propagates misfolding when transferred to transgenic mice or cell cultures^3,4^.

Though sharing a similar general process of seeding and assembly of αSyn, the etiology and clinical manifestations of α-synucleinopathies are different, as are the roles of αSyn as both an effector of neurotoxicity and as a mediator of the pathogenicity and disease progression. These differences are manifest in macroscopic structural differences in the deposited aggregates^5,6^, ascribed to different conformational polymorphs of fibrillar αSyn^6^. Further suggesting a connection between αSyn fibril structure and disease, in vitro-amplified αSyn fibrils differ in their seeding and self-propagation behavior in vivo, inducing polymorph-specific pathology and neurotoxic phenotypes^6–12^.

In addition to the putative structural differences attributed to different α-synucleinopathies, αSyn might adopt at least two structural polymorphs within a single disease reported for MSA, which might further vary between clinical cases^13^. Moreover, there could be more heterogeneity among PD than MSA fibrils, which may explain the greater variety of disease phenotypes in PD^6,14^. Finally, differences in the structure of αSyn fibrils might arise from different post-translational modifications, in particular phosphorylation and ubiquitination, and thus could account for the differing pathogenesis and disease progression within a single α-synucleinopathy^8^.

Thus, to gain insight into the connection between the molecular structure of αSyn fibrils and the ability to seed endogenous αSyn in recipient cells, we fibrilized αSyn by protein misfolding cyclic amplification (PMCA) using brain extracts of patients pathologically confirmed with PD and MSA, solved their cryo-EM structures, and evaluated their seeding potential in mouse primary oligodendroglial cultures. The structural analysis and cellular experiments were performed on the same sample to enable a direct connection between molecular structure and cellular seeding and avoid the influence of variations in the aggregation process. The combined analysis identifies the quaternary structure of αSyn fibrils as a vital factor in the seeding of αSyn pathology.

## Results

### MSA-PMCA αSyn fibrils are more active in oligodendroglia than PD-PMCA fibrils

Recombinant αSyn can directly nucleate and form fibrils in vitro, but the timescales are very long and high concentrations are required unless seeds are used, or aggregation-accelerated mutations are present^15^. On the other hand, PMCA is a well-established procedure that proved to amplify aggregates from blood^16^ and cerebrospinal fluid^17^ reliably and was also successfully applied for an αSyn anti-aggregating drug screening^15^. We therefore seeded fibril formation of recombinant αSyn through the addition of PMCA-products, which were previously generated from the homogenized brain tissue of a PD and a MSA patient (PD patient #1 and MSA patient #1 in ref. ^6^). We selected patients PD1 and MSA1 from our previous study (ref. ^2^) since they had similar disease duration but were most different in their NMR-derived hydrogen-deuterium exchange profile (Fig. 5b. in ref. ^2^). In our previous work^6^, Western blot analysis with an αSyn-specific antibody and fluorescence measurements using the amyloid-binding dye thioflavin-T (ThT) showed that we successfully amplified αSyn aggregates from the brain extracts. Furthermore, control PMCA experiments with brain extract from an individual, in which an α-synucleinopathy was excluded, did not amplify αSyn aggregates. In addition, hydrogen-deuterium exchange coupled to NMR spectroscopy showed that the brain tissue-amplified αSyn fibrils (further termed PD- and MSA-PMCA αSyn fibrils) differ in structural integrity properties^6^.

**Fig. 1:**
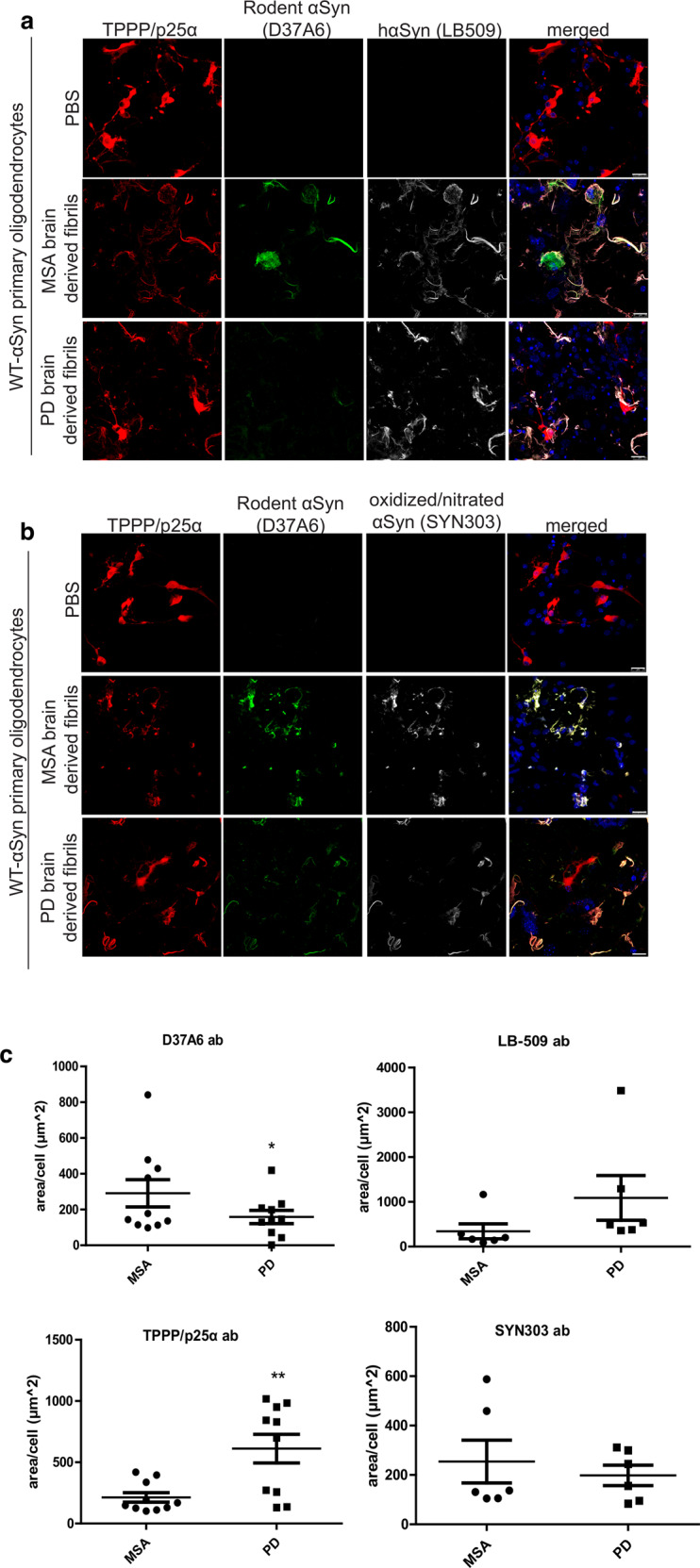
Human αSyn fibrils amplified from a MSA patient are more potent in recruiting the endogenous oligodendroglial αSyn and evoking a redistribution of TPPP/p25α protein in mouse primary oligodendroglial cultures, compared to those amplified from a PD patient. Both MSA-PMCA and PD-PMCA fibrils form pathological αSyn species (oxidized/nitrated αSyn, SYN303 ab). **a**, **b** Representative immunofluorescence images of mouse primary oligodendrocytes treated with 0.5 μg of MSA-PMCA and PD-PMCA fibrils for 48 h (or PBS as control) using antibodies against TPPP/p25α (red), endogenous rodent αSyn (D37A6 antibody, green), human αSyn (LB509 antibody, gray in **a**), oxidized/nitrated αSyn (SYN303 antibody, gray in **b**) and DAPI (shown in blue) staining as a nuclear marker. Scale bar: 25 μm. **c** Quantification of the endogenous rodent αSyn (upper left), human αSyn (upper right), TPPP/p25α (lower left), and oxidized/nitrated αSyn (lower right) protein levels in mouse primary oligodendrocytes, measured as μm^2^ area surface/cell following their treatment with 0.5 μg of MSA-PMCA and PD-PMCA fibrils for 48 h. Data are expressed as the mean ± SE of three independent experiments with duplicate samples/conditions within each experiment; *p < 0.05; **p < 0.01, by Student’s unpaired *t*-test.

To gain insight into their differences in the cellular activity of the PD- and MSA-PMCA αSyn fibrils, we added each fibril sample to differentiated murine primary oligodendroglial cultures. The distinct profiles of PD- and MSA-PMCA fibril strains were further validated by the differential pathology-related responses observed in these cultures upon inoculation with PMCA fibrils. In particular, the experiments show that MSA-PMCA fibrils display higher potency in seeding the endogenous oligodendroglial αSyn and promoting the redistribution of the oligodendroglial-specific phosphoprotein TPPP/p25α from the myelin sheath to the cell soma, as compared to PD-PMCA fibrils (Fig. 1). Both events play an essential role in the cascade of events leading to oligodendroglial dysfunction and neuronal demise underlying MSA pathology.

### MSA-PMCA and PD-PMCA αSyn fibrils share a common protofilament fold

**Fig. 2:**
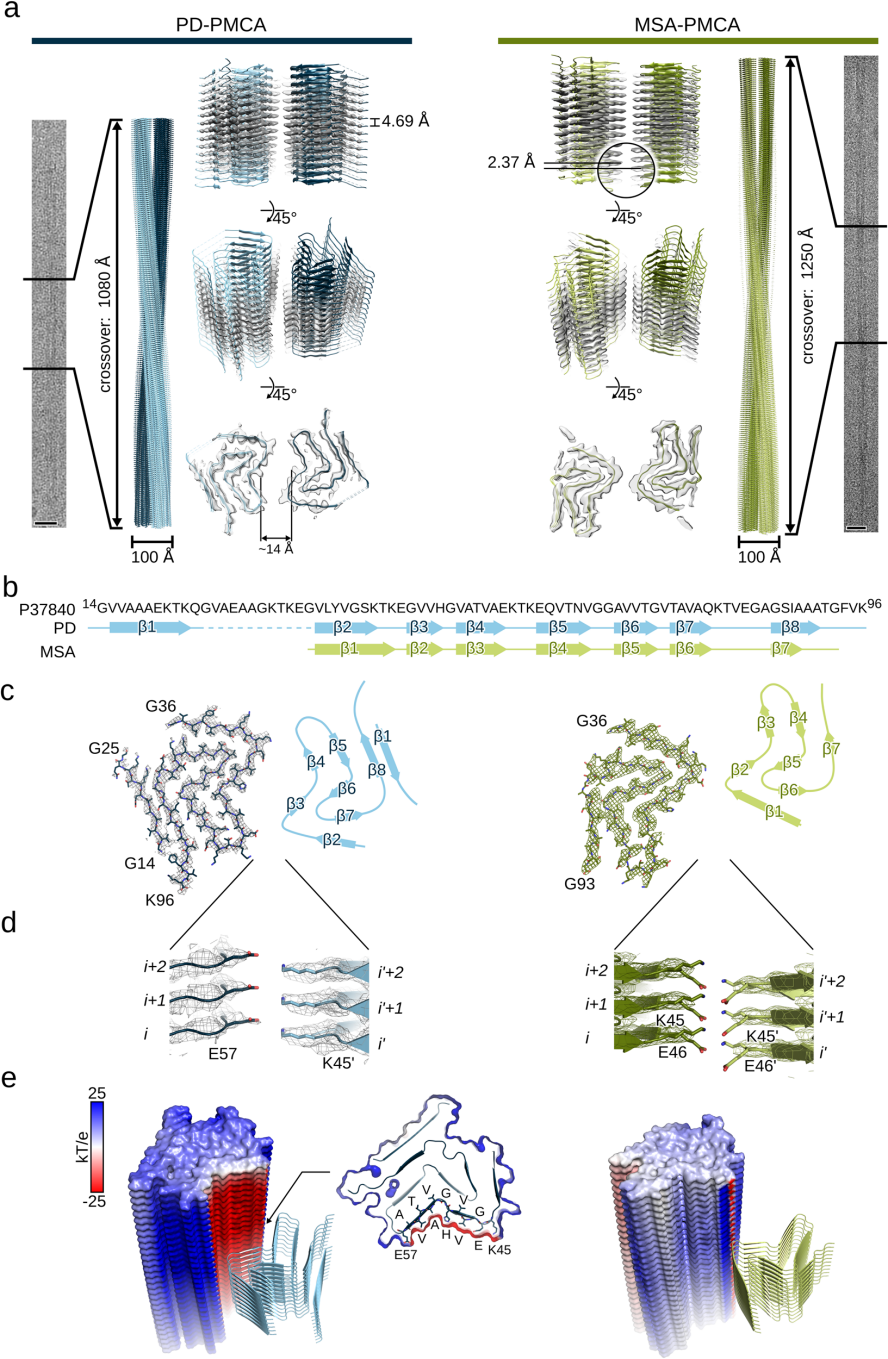
Cryo-EM structures of αSyn PMCA fibrils seeded from brain homogenates. **a** Cryo-EM structures of PD-PMCA (left) and MSA-PMCA (right) αSyn fibrils. From the outside to the inside, the panels show extracts from representative micrographs, a full cross over (180° turn) of the reconstructed fibril, with two protofilaments colored in different shades of blue (PD-PMCA) or green (MSA-PMCA), and semitransparent surfaces overlaid with their atomic models viewed from different angles. Scale bars, 20 nm. **b** Amino acid sequence of αSyn (from G14 to K96; based on UniProt: P37840) with a schematic depiction of the secondary structure of the protofilament fold. β-strands are shown as arrows and numbered from β1 to β8 (for PD) or β7 (for MSA), respectively. The region from V26 to E35 was not resolved (indicated by a dashed line). **c** Top view onto two opposite subunits of the reconstructed PD (left; colored in shades of blue) and MSA (right; colored in shades of green). One protofilament is shown as a mesh-stick model, the other schematically depicted by its secondary structure matching the assignment in **b**. **d** Close-up view of the protofilament interface, with interface amino acids shown as stick models. **e** The calculated electrostatic potential was mapped onto the surface of one protofilament and colored according to the color scale on the left. The central subunits of the other protofilament are shown as cartoon model. Cross-sections are shown as a surface-cartoon model with amino acids forming the central negatively charged cavity in PD-PMCA αSyn (from K45 to E57) labeled explicitly.

To elucidate why primary oligodendroglia respond differently to PD- and MSA-PMCA αSyn fibrils, we determined their 3D structure by cryo-EM. The 3D structures of the PD- and MSA-PMCA αSyn fibrils were determined using the same samples that induced the differential response in the oligodendroglia. We observed a single dominant fibril type in the micrographs for both fibril samples. The measured crossover distances were ~1000 Å and ~1200 Å for the PD- and MSA-PMCA αSyn fibrils, respectively (Fig. 2a, b and Fig. S1). In the case of the MSA-PMCA αSyn fibrils, we also observed non-twisted fibrils (Fig. S1), which were considered preparation artifacts from interactions with the air-water interface^18^.

The 3D structures of PD and MSA-PMCA αSyn fibrils were determined to a resolution of 3.3 Å and 3.0 Å, respectively, based on the Fourier shell correlation 0.143 criterion (Tables 1, 2, Fig. 2, and Fig. S2). In the case of the MSA-PMCA αSyn fibril, the local resolution estimation revealed a pronounced heterogeneity with a resolution of ~2.9 Å in the inter-protofilament interface and >4.0 Å at the periphery of the fibril (Fig. S3). In contrast, for the PD-PMCA fibril, the local resolution estimates were more homogenous and, hence, the reconstructed map revealed precise side-chain densities. This suggests that the overall fold of the PD-PMCA αSyn fibril tends to be more rigid than the MSA-PMCA αSyn fibril.

**Table 1:** Cryo-EM structure determination statistics.

	PD-PMCA αSyn	MSA-PMCA αSyn
Data collection		
Microscope	Titan Krios G2	Titan Krios G2
Voltage [keV]	300	300
Detector	K3	K3
Magnification	81,000	81,000
Pixel size [Å]	1.05	1.05
Defocus range [µm]	−0.7 to −2.9	−0.7 to −2.9
Exposure time [s/frame]	2.2	2.2
Number of frames	40	40
Total dose [e^−^/Å^2^]	42.74 (1.07 e^−^/Å^2^/frame)	43.05 (1.08 e^−^/Å^2^/frame)
Reconstruction		
Micrographs	6,780	4,474
Box width [pixels]	200 (1.05 Å/pixel)	250 (1.05 Å/ pixel)
Inter-box distance [pixels]	18	50
crYOLO^a^ picked segments (no.)	2,223,091	1,848,677
Final segments [no.]	23,937	25,692
Final resolution [Å] (FSC = 0.143)	3.30	3.02
Applied map sharpening B-factor [Å^2^]	−112.5	−101.8
Symmetry imposed	C2	C1
Helical rise [Å]	4.68	2.37
Helical twist [°]	−0.78	179.66

^a^ref. ^61^

**Table 2:** Model building statistics.

	PD-PMCA αSyn	MSA-PMCA αSyn
Starting model [PDB code]	6SSX^a^	6SST^a^
Model composition		
Non-hydrogen atoms	497	389
Protein residues	73	58
RMS deviations		
Bond lengths [Å]	<0.05	<0.05
Bond angles [°]	2.01	1.68
Validation		
MolProbity score	2.25	2.02
Clashscore	17.17	14.41
Poor rotamers [%]	0.00	0.00
Ramachandran plot		
Favored [%]	91.30	94.64
Allowed [%]	8.70	5.36
Disallowed [%]	0.00	0.00
Model deposition		
PDB code	7OZG	7OZH
EMD code	13123	13124

^a^ref. ^20^

The reconstructed maps of both αSyn fibril types show a clear β-strand separation along the helical axis and two intertwined protofilaments (Fig. 2a, b). For the PD-PMCA fibrils, the protofilaments are related by C2 symmetry (helical rise = 4.68 Å and twist = −0.78°). In contrast, the protofilaments are related by an approximate pseudo 2_1_ screw symmetry (helical rise = 2.37 Å and twist = 179.66°) in MSA-PMCA fibrils. Therefore, the refined crossover distances are in excellent agreement with the measurements from the micrographs. In both cases, the fibril width is ~100 Å. Due to the lack of twist^13,18^, the 3D structure of the non-twisted MSA-PMCA αSyn fibrils could not be solved.

The cryo-EM structures reveal that the protofilament folds of the PD- and MSA-PMCA αSyn are highly similar. The PD-PMCA fold extends from G14 to K96 and is composed of eight β-sheets, from which β2 to β8 are connected by a continuous backbone chain and β2/β3, β4/β5, and β6/β7 form a triple-stacked *L*-shaped core (Fig. 2c, d). No apparent densities were found for the N-terminus from M1 to E13, the region from V26 to E35, and the C-terminus beyond K96, suggesting that these regions are more flexible.

The MSA-PMCA fold extends from G36 to G93 and comprises seven β-sheets, creating a continuous backbone chain and, similar to the PD-PMCA αSyn fibrils, the β-sheets form the same *L*-shaped core (Fig. 2a, b). In contrast to the PD-PMCA αSyn fibrils, no backbone densities were found for the first 38 amino acids, similar to other structures of αSyn lacking the same region^19^.

### Distinct quaternary arrangement of MSA-PMCA and PD-PMCA αSyn fibrils

While the MSA-PMCA and PD-PMCA αSyn fibrils share a common protofilament fold, they display different quaternary arrangements (Fig. 2a, c). In the PD-PMCA αSyn fibril, two salt-bridges between K45 and E57' and between E57 and K45', harbored on two opposite subunits *i* and *i’* form the inter-protofilament interface (Fig. 2d). In contrast, the inter-protofilament interface of the MSA-PMCA fibrils is formed by a sophisticated salt-bridge network, in which K45 from subunit *i* interacts with E46 on subunit *i* + *1* and E46' on subunit *i*′ + *1* (Fig. 2d). Additionally, E46 on subunit *i* interacts with K45' from subunit *i’*. The intact integrity of the network explains why the subunits are arranged in a staggered manner, resulting in an approximate pseudo 2_1_ screw symmetry of the MSA-PMCA αSyn fibrils.

The distinct inter-protofilament interfaces imprint different structural properties onto the PD-PMCA and MSA-PMCA αSyn fibrils. This becomes apparent when analyzing the electrostatic surface potential. For example, the PD-PMCA fibril is positively charged on the exterior surface, but the inter-protofilament cavity is negatively charged (Fig. 2e and Fig. S4). The latter is notable as the amino acids forming the cavity are not negatively charged except E46 and E57. In contrast, the inter-protofilament cavity is positively charged in the MSA-PMCA fibril. In addition, the exterior surface of the MSA-PMCA αSyn fibril is less positively charged than the PD-PMCA αSyn fibril surface, particularly at the now almost-neutral C-terminus, which the absence of the N-terminus may explain.

### PMCA fibrils reveal similarities to in vitro aggregated αSyn fibrils

**Fig. 3:**
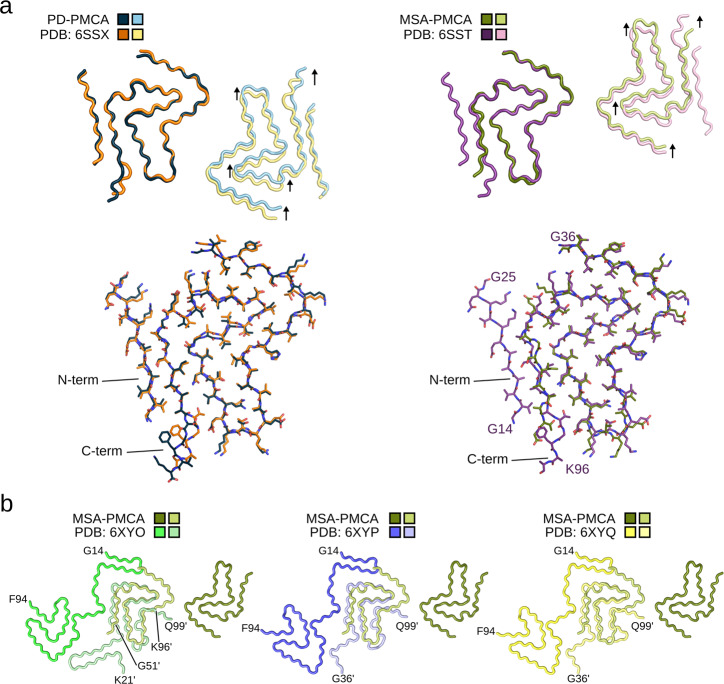
Amplified αSyn fibrils reveal a common protofilament fold. **a** Overlay of the PD-PMCA and the MSA-PMCA αSyn fibril structures onto two opposite subunits of recombinant in vitro aggregated αSyn fibrils with PDB IDs 6SSX (termed “polymorph 2A”) and 6SST (termed “polymorph 2B”)^20^, respectively. Arrows mark relative shifts between the in vitro αSyn fibrils and the PD/MSA-PMCA αSyn fibrils (displayed in light colors) after superimposing the Cα atoms of the opposite subunits (displayed in dark colors). Below, the superposition of single protofilaments is shown, highlighting changes in the C-terminal structure. **b** Overlay of the MSA-PMCA αSyn fibril structure determined in the current study (labeled “from MSA”) onto structures determined for αSyn fibrils extracted from the brains of individuals with MSA. The ex vivo MSA fibril structures (PDB IDs 6XYO, 6XYP, and 6XYQ)^13^ are colored in shades of green, blue, and yellow, respectively, with amino acids from subunit B labeled with an additional prime.

Next, we compared our αSyn structures to previously solved αSyn structures and used the Cα root mean square deviation (Cα RMSD) to measure structural similarity. Our PD-PMCA αSyn structure is similar to a fibril structure determined for recombinant wild-type αSyn, which was aggregated in the absence of brain homogenates and was named polymorph 2A^20^ (Fig. 3a). Comparison of the polymorph 2A with the PD-PMCA αSyn fibril structure results in Cα RMSDs of 1.03 Å and 1.33 Å considering one or both protofilaments, respectively (Table S1). Only minor structural deviations were present at the C-terminus from T92 to K96 (Fig. 3a). Although the protofilament fold and inter-protofilament interface are almost identical, the helical rise and twist are different (Table S1), resulting in a lateral displacement of the protofilaments relative to each other (Fig. 3a).

The MSA-PMCA αSyn structure is similar to the in vitro αSyn fibril structure referred to as polymorph 2B^20^ with Cα RMSDs of 1.47 Å and 1.74 Å considering the resolved amino acids G36 to G93 of one or both protofilaments, respectively (Fig. 3a and Table S2). In contrast to polymorph 2B, however, the N-terminal region from G14 to G25 is not visible in the MSA-PMCA structure, suggesting that the N-terminus tends to be more flexible. In addition, the helical rise and twist differ between the MSA-PMCA and the polymorph 2B structure (Table S2).

### Conserved backbone shape of PMCA fibrils and ex vivo αSyn fibrils

Cryo-EM structures of αSyn fibrils extracted using sarkosyl from the brains of MSA-confirmed patients have been previously reported^13^. Two types of αSyn fibrils were observed in these preparations and their relative abundance varied. For three patients, predominantly, either type I or type II fibrils were detected, while in the other two patients, both fibril types were found. The ex vivo αSyn fibrils differ in their inter-protofilament interfaces but share an identical three-layered *L*-shaped fold^13^, which is similar to that observed in the MSA-PMCA αSyn fibril. Indeed, the anti-parallel superposition reveals a similar structural backbone shape in αSyn fibrils purified from MSA patient’s brain and the MSA-PMCA αSyn fibrils seeded from the brain homogenate of an MSA patient: in both a triple-stacked *L*-shape is present (Fig. 3b), but with opposite sequence direction.

Next, we compared our amplified αSyn structures to other cryo-EM structures from Lövestam et al.^18^, for which the αSyn was aggregated in the presence of brain extracts of MSA patients but without using PMCA (Table S1, S2). Superposition of one protofilament strand yields Cα RMSDs <2 Å, but superimposing two opposite protofilament strands yields Cα RMSDs >10 Å. This indicates that the global symmetry, rise, twist, and, thus, helical organization, but not the core protofilament fold are different between αSyn fibrils, which were amplified from brain homogenates of two different MSA patients using different amplification protocols (Tables S1 and S2). MSA-associated fibrils, either purified by sarkosyl extraction or generated through seeding with brain homogenate, thus share a common protofilament core.

## Discussion

The misfolding and aggregation of αSyn is the pathological hallmark of both PD and MSA^21,22^. Over recent years, αSyn fibrils prepared in various ways have been suggested as potential toxic species with clinical relevance for PD and MSA^23,24^. However, a firm understanding of how αSyn fibrils associated with PD or MSA hamper cellular function has remained elusive. Here, we amplified αSyn fibrils from brain extracts of patients pathologically confirmed with PD or MSA, determined their 3D structures by cryo-EM, and evaluated their potential to seed αSyn-related pathology in oligodendrocytes. Our results show that αSyn fibrils amplified from MSA and PD brain homogenate share a common protofilament fold, but differ in their quaternary structure and their ability to seed endogenous αSyn in mice primary oligodendroglial cultures.

The 3D structures of the PD- and MSA-PMCA αSyn fibrils revealed major differences between both types in their inter-protofilament interfaces, their adopted helical arrangement, and the lacking N-terminal region in the MSA-PMCA αSyn fibril (Fig. 2). Notably, the determined structures are very similar to two previously solved αSyn structures, namely polymorphs 2A and 2B, originating from αSyn aggregated in vitro under continuous-shaking conditions and in the absence of brain homogenate^20^ (Fig. 3). However, polymorphs 2A and 2B were observed next to each other and originate from an identical preparation^20^, suggesting that both polymorphs are thermodynamically-stable αSyn aggregates under particular conditions.

In the present study, we also used recombinant αSyn as the substrate for PMCA. However, we obtained a single main conformation in independent experiments, either with seeds from PD or MSA-diagnosed brains (Fig. 2). PMCA is based on the propensity of prions and prion-like proteins to act like seeds and replicate in an autocatalytic process, thereby converting recombinant protein as a substrate into amyloid fibrils. Indeed, the PMCA technique successfully amplifies and detects misfolded prion proteins^25,26^, and several lines of evidence suggest prion-like features for αSyn^22,27,28^. Considering that the identical and extensively validated PMCA procedure was used to amplify αSyn seeds from either PD or MSA^6^ yields different αSyn structures, suggests that the brain homogenates must contain structurally different αSyn seeds that served as the starting point for PMCA-induced fibrilization leading to the fibrils described herein.

The key finding from our study is that the PD- and MSA-PMCA αSyn fibrils, with their similar protofilament fold but distinct quaternary arrangement, have different activity when added to mouse primary oligodendroglial cultures: the MSA-PMCA fibrils are more potent in recruiting the endogenous oligodendroglial αSyn and evoking a redistribution of TPPP/p25α protein when compared to the PD-PMCA fibrils (Fig. 1). Both events are characteristic of MSA^29^. Thus, under physiological conditions, TPPP/p25α is predominant in myelin sheaths^30^, but under MSA-related pathological conditions, TPPP/p25α relocates to the oligodendrocyte soma^31^. Notably, TPPP/p25α not only co-localizes with filamentous αSyn^32^ but also fosters further aggregation of αSyn into filamentous aggregates^33^. The C-terminus of αSyn fibrils has been identified as the binding epitope for TPPP/p25α^33–35^, but an atomic picture of how TPPP/p25α binds to filamentous αSyn has not been realized yet. Considering the wide range of αSyn fibril structural polymorphism^36^ (Fig. S5) and that neither in our (Fig. 2) nor any structurally related fibril (Fig. 3a; Table S1 and S2) the C-terminus was resolved, clarification of the full-length αSyn structure under pathological conditions might be necessary to elucidate the underlying structure-activity relationship fully. These observations suggest that some features from the MSA seeds (e.g. the filament interface or the disordered N-terminus) have been transferred to the amplified fibrils, leading to a more MSA-like response in oligodendroglial cells than PD-PMCA fibrils. Alternatively, and beyond the scope of this study, one might also consider an indirect effect of αSyn fibrils on TPPP/p25α redistribution and co-localization.

A fundamental difference between PD and MSA is that in the case of PD αSyn aggregates are predominantly present in dopaminergic neurons, whereas MSA is associated with αSyn inclusions within oligodendrocytes^37,38^. Peng et al. showed that the intracellular environments of both neurons and oligodendrocytes determine how the same misfolded αSyn seeds develop into different aggregates^5^. While dopaminergic neurons are directly associated with reward-motivated behavior and motor control, oligodendrocytes are essential for the long-distance saltatory conduction of neuronal impulses as they enwrap central nervous system axons with the myelin sheath, a lipid enriched multilayer membrane^39,40^. Although the lipid content and composition of oligodendrocytes is still unknown, these numbers are well known for the myelin sheath^41,42^, which is extremely rich in lipids (~80% of its dry weight)^39,40^. Previous studies revealed the αSyn N-terminus is essential for lipid-binding^43^ and fatty acid-induced oligomerization^44^. Further biophysical experiments suggest that αSyn-lipid interactions are predominantly driven by electrostatic interactions between charged N-terminal residues and the charged head groups of phospholipids^45^, which comprise ~26% of all lipids found in central nervous system myelin^41^. Thus, the αSyn N-terminus may interact with these phospholipids during fibril aggregation and seed formation in oligodendrocytes, which may hamper the stabilization of the folded N-terminus in these cells in turn. One could then speculate whether the disordered N-terminus observed in the MSA-PMCA structure is a feature inherited from the seed, although no lipids were present during in vitro fibrilization^6^.

There are two distinct mechanisms by which homogeneous seeds added to monomer solutions can induce aggregation: either the seeds can be elongated through monomer addition, or the seeds can nucleate new fibrils on their surface (secondary nucleation). Elongation mechanisms necessarily perpetuate the structure of the seeds onto monomers, with the lamination of successive cross-beta layers. On the other hand, secondary nucleation of αSyn relies on transient interactions between monomer and fibril surface^46^, with the monomer less closely coordinated with the protofilaments and thus potentially able to adopt a conformation differing from the seeds, though energetic considerations can favor mimicking of the seed packing and morphology^47^. Therefore, when seeds are amplified in vitro, αSyn can sometimes form fibrils of a different structure under different solution conditions. Under conditions conducive to elongation, seeds imprint their structural properties onto fibrils growing by recruiting soluble αSyn molecules^23,48^. In contrast, it has been shown that in conditions favoring secondary nucleation, the propagation of seed structural properties decreases^48^. Consequently the structure of fibrils, even when formed in the presence of seeds, may be influenced by differing environmental conditions, such as temperature^49^, the concentration of seeds^48,50^, pH^49,51^, salt concentration^52^, and the presence of surfaces for nucleation such as air-water interfaces^53^ or lipids^54^. We note, however, that although the MSA-PMCA structure determined in the current study does not fully replicate the previously identified structures of αSyn fibrils extracted with sarkosyl from the brain samples of MSA patients^18^, the PMCA-derived structures can be partially aligned in an antiparallel arrangement with the triple-stacked *L*-shape core region comprising about 40 residues (Fig. 3b). Therefore, it is conceivable that this matching 40-residue core region biases a monomer into a fibril compatible conformation by forming an anti-parallel cross-beta sheet and thus acts as a seed for the PMCA structures observed in the present work and in Lövestam et al.^13,18^. In addition, we note that our previous data revealed some structural heterogeneity between fibrils amplified from five MSA and five PD patients, such that one might even assume that fibrils may be, to at least some extent, patient-specific^6^. Consistent with this hypothesis, two types of αSyn fibrils were observed in different MSA patients and their relative abundance varied from patient to patient and between different brain regions^13,18^.

Here we establish a connection between the structure and cellular seeding activity of αSyn fibrils amplified from MSA and PD brain-derived seeds. Specifically, the quaternary structure of αSyn fibrils influences the seeding of αSyn pathology inside oligodendroglia. Our study thus makes an essential contribution to ongoing research efforts into unraveling the importance of αSyn fibril structure for cellular seeding, the spreading of αSyn pathology in the brain and thus the clinical manifestations in different synucleinopathies.

## Methods

### Preparation of brain extracts

Ethical approval to access and work on the human brain tissues was given by the Human Research Ethics Committee of the University of New South Wales. Following approvals, brain tissues from one PD patient (PD1 in ref. ^6^) and one MSA patient (MSA1 in ref. ^6^), respectively, were received from the Sydney Brain Bank at Neuroscience Research Australia, which is supported by The University of New South Wales and Neuroscience Research Australia. Patient PD1, male, died at age 79; sample was taken with postmortem delay of 17 h; cause of death was acute myocardial infarction, and disease duration was 7 years. Patient MSA1, male, died at age 82; sample was taken with postmortem delay of 8 h; cause of death was cardiorespiratory failure, and disease duration was 7 years. Human amygdalas were sonicated with Vibra-cells (Sonics, Newtown, CT, USA) to 10% weight/volume (w/v) solution with homogenizing buffer (1% Triton X-100, Protease Inhibitor Cocktail in PBS). Sonicated samples were centrifuged at 3000 *g* for 40 s. Protein concentrations in supernatants were determined by the bicinchoninic acid assay (Pierce, Rockford, IL, USA).

### Recombinant αSyn preparation

N-terminally acetylated αSyn was obtained by co-transfection of *E. coli* BL21 (DE3) cells with pT7-7 plasmid encoding for human αSyn (kindly provided by the Lansbury Laboratory, Harvard Medical School, Cambridge, MA) and *S. pombe* NatB acetylase complex^37^ using pNatB plasmid (pACYCduet-naa20-naa25, Addgene, #53613, kindly provided by Dan Mulvihill). The mutant protein αSyn-T54CA90C was constructed using the QuikChange site-directed mutagenesis kit (Stratagene), and the introduced modifications were verified by DNA sequencing.

For αSyn expression and purification, transformed BL21 (DE3) cells were grown at 37 °C in LB medium to an OD_600_ of 0.8 and shifted to 25 °C adding 0.5 mM IPTG for protein expression overnight. Cells were harvested by centrifugation on a Beckman Coulter Avanti JXN-26 centrifuge with a JLA-8.1 rotor at 12,000 *g* for 15 min at 4 °C. The obtained cell pellet was lysed by French press (Avestin EmulsiFlex-C3) in 20 mL lysis buffer (10 mM Tris-HCl, pH 8, 1 mM EDTA, 1 mM PMSF) per 1 L cell culture. The cell lysate was heated up to 96 °C in a water bath and incubated at this temperature for 30 min. The supernatant was collected by centrifugation (Beckman Coulter, JA-25.5 rotor, 22,000 *g*) at 4 °C for 30 min. Streptomycin sulfate was added to the supernatant at a final concentration of 10 mg mL^−1^ and incubated at 4 °C for 15 min. The supernatant was collected by centrifugation (JA-25.5, 30 min, 22,000 *g*) and ammonium sulfate was added to a final concentration of 360 mg mL^−1^ followed by incubation at 4 °C for 15 min. After a final centrifugation step, the protein pellet was obtained and dialyzed against 25 mM Tris-HCl, pH 7.7 overnight. The dialysate was applied to an anion exchange column (GE Healthcare, Mono Q 5/50 GL) and eluted with 25 mM Tris-HCl, pH 7.7, 2.0 M NaCl using a salt gradient from 0 to 1 M. The αSyn fraction eluted at 300 mM NaCl. High αSyn purity and buffer exchange was achieved by a final size exclusion run (GE Healthcare, Superdex 75 10/300 GL) with 50 mM HEPES, 100 mM NaCl, pH 7.4, 0.02% NaN_3_ on a GE Healthcare Äkta pure system.

### PMCA

Equipment for PMCA, which includes the microplate horn (#431MPX), a sound enclosure (#432MP) and a thermoelectric chiller (#4900), was purchased from Qsonica (Qsonica, Newtown, CT, USA). PMCA was carried out with recombinant αSyn and tissue homogenates. αSyn monomers were prepared at 2.5 μM concentration in the conversion buffer (1% Triton X-100 in PBS) and 50 μL were transferred into PCR tubes that contained 2.5 μg of brain homogenates. Three Teflon beads were placed in the PCR tubes before adding the mixture. For PMCA, the samples were subjected to 48 cycles of 20 sec sonication (amplitude 1%) and 29 min 40 sec incubation at 37 °C ^55^. PMCA product was analyzed by Western blot using the αSyn-specific antibody from BD Transduction, USA (#610787) at a dilution of 1:1500.

0.5% (w/w) PMCA product was added to 250 μM αSyn stock solution (50 mM HEPES, 100 mM NaCl, pH 7.4, 0.02% NaN_3_) and initially water bath sonicated for 10 min. This mixture was aggregated for 5 days under quiescent conditions in 1.5 mL Eppendorf cups in a ThermoScientific Heratherm incubator.

### Primary oligodendroglial cultures

Mixed glial cultures generated from P0 to P3 neonatal wild-type mice were maintained in full DMEM for 10 to 14 days until a monolayer of astrocytes on the bottom and primary oligodendroglial progenitor cells (OPCs) with loosely attached microglia on the top, were apparent. The separation of OPCs was achieved initially with the removal of microglia, by shaking in 200 rpm for 1 h in 37 °C and then with continuous shaking under the same conditions for 18 h, as previously described^56^. Afterwards, isolated cells were platted on poly-D-lysine-coated coverslips (P7405, Sigma-Aldrich, USA) with a density of 80,000 cells/mm^2^ and maintained in SATO medium (284369) supplemented with Insulin-Transferrin-Selenium solution (41400045, ITS- Gibco, Invitrogen, Carlsbad, CA, USA), 1% penicillin/streptomycin and 1% horse serum (H1138; Sigma-Aldrich, St. Louis, MO, USA) for 4 days. αSyn fibrils (final concentration 0.5 µg/mL culture medium/well) amplified from human MSA and PD brains were added to TPPP/p25α-positive mature differentiated oligodendrocytes for 48 h and then cells were fixed and preceded for immunofluorescence analysis. All experimental procedures were approved by the Ethics Committee for the Use of Laboratory Animals in the Biomedical Research Foundation of Athens.

### Immunocytochemistry and confocal microscopy

At 48 h post patient-PMCA fibril addition, cells were fixed with 4% paraformaldehyde for 40 min, blocked in 10% normal goat serum containing 0.4% Triton X-100 for 1 h at room temperature, and incubated with antibodies against the human (LB509), the rodent (D37A6), or the oxidized/nitrated (Syn303) αSyn and the oligodendroglial phosphoprotein TPPP/p25α (concentration of 0.2 mg/mL and diluted by 1:400, kind gift from Dr. Poul Henning Jensen, Aarhus University, Denmark) overnight at 4 °C. Antibody concentrations of LB509 and SYN303 were 1 mg/mL and a dilution of 1:1000 was used for the experiments. For antibody D37A6 a dilution of 1:400 of the stock solution was used. Images were obtained using a Leica TCS SP5 confocal microscope combined with a dual (tandem) scanner. All confocal images were obtained under equivalent conditions of laser power, pinhole size, gain, and offset settings between the groups. ImageJ (v2.0.0) software was used to quantify relative protein levels expressed as % area coverage, normalized to the p25α + cells/field.

### Cryo-EM grid preparation and imaging

Sample volumes of 3.5 μl were applied to freshly glow-discharged R3.5/1 holey carbon grids (Quantifoil) and vitrified using a Mark IV Vitrobot (Thermo Fischer Scientific) operated at 100% rH and 20 °C. Micrographs were collected with a Titan Krios transmission-electron microscope operated at 300 keV accelerating voltage at a nominal magnification of 81,000× using a K3 direct electron detector (Gatan) in non-superresolution counting mode, corresponding to a calibrated pixel size of 1.05 Å on the specimen level. In total, 6780 PD-PMCA and 4474 MSA-PMCA micrographs with defocus values in the range of −0.7 μm to −2.9 μm were recorded in movie mode with 2.2 sec acquisition time. Each movie contained 40 frames with an accumulated dose of ~43 electrons per Å^2^. The resulting dose-fractionated image stacks, containing all frames 1–40, were subjected to beam-induced motion correction using MotionCor2^57^, prior to helical reconstruction. Estimation of contrast transfer function parameters for each micrograph was performed using CTFFIND4^58^. Subsequently, PD-PMCA and MSA-PMCA fibrils were reconstructed using RELION-3.1^59^, following the helical reconstruction scheme^60^.

### Helical reconstruction of PD-PMCA fibrils

crYOLO^61^ was used for the selection of 76,170 fibrils in the data set, from which 2,223,091 segments were extracted using a 19 Å inter-box distance. Using RELION-3.1^59^, maximum-likelihood two-dimensional (2D) reference-free classification, and 3D classification were performed on an unbinned data set (1.05 Å/px, 200 px box size); a cylinder with white noise added using EMAN2^62^ was used for the initial reference.

We used the CHEP algorithm^63,64^ with *k*-means clustering on the results of 2D classification to identify and group fibrils by overall conformation (*k* = 3), the first two clusters were joined and used for further processing. The algorithm associates segments extracted from a single helical fibril and clusters each fibril together with similar fibrils, based on the similarity of their segments within the classification. On a single-subunit level the signal-to-noise ratio is often not enough to distinguish between two similar classes, especially for some projections where the conformations appear more similar.

After iterative classification steps, 23,937 particles were selected for 3D auto-refinement, beam tilt refinement, CTF refinement, and reconstruction in RELION-3.1^59^. During the post-processing step in RELION, the map was masked with a soft mask and a B-factor of −112 Å^2^ was applied, and the resolution was estimated as 3.3 Å based on the gold-standard Fourier shell correlation 0.143 criterion (Fig. S2a). The helical geometry was then applied to the map, which was then re-sharpened using VISDEM^65^. Local resolution was determined using RELION-3.1^59^.

### Helical reconstruction of MSA-PMCA fibrils

crYOLO^61^ was used for the selection of 50,794 fibrils in the data set, from which 1,848,677 segments were extracted using a 50 Å inter-box distance and RELION-3.1^59^ was used for reconstruction. To exclude non-twisted and irregularly twisted segments, we initially performed several rounds of 2D classification on a downscaled data set (2.1 Å/px, 500 px box size). For 3D classification we (re-)extracted 91,552 segments without downscaling (1.05 Å/px, 250 px box size).

Starting from a featureless cylinder filtered to 60 Å, we performed a 3D classification with one class and *T* = 3, followed by a 3D classification with one class and *T* = 4, after which the two protofilaments were separated and first backbone features became visible. Subsequently, another round of 3D classification with five classes and *T* = 4 yielded one class showing clear backbone features. This class was selected (25,692 particle segments) for further rounds of 3D classification (*k* = 1) with step-wise adjusting the *T*-value from 4 to 8 after which separation of β-strands along the Z-axis and the approximate 2_1_ screw symmetry between the two protofilaments became visible. From here on, we performed multiple rounds of 3D auto-refinement until no further improvement of the map was observed. Assuming a left-handed twist, the helical twist and rise converged to 179.66° and 2.37 Å, respectively, in agreement with the predominant crossover distances measured on the motion-corrected cryo-EM micrographs. Finally, post-processing with a soft-edged mask and an estimated sharpening *B*-factor of −101 Å^2^ yielded post-processed maps. The resolution was estimated from the value of the FSC curve for two independently refined half-maps at 0.143 (Fig. S2b). The helical geometry was then applied to the map, which was then re-sharpened using VISDEM^65^. Local resolution was determined using RELION-3.1^59^.

### Atomic model building and refinement

PDB entries 6SSX and 6SST^20^ were used for an initial model for PD-PMCA and MSA-PMCA fibrils, respectively. Subsequent refinement in real space was conducted using PHENIX^66,67^. For PD-PMCA, the final refined protofilament subunit had an RMSD of 0.64 Å to PDB 6SSX. As for MSA-PMCA, the final refined protofilament subunit had an RMSD of 0.95 Å to PDB 6SST.

### Determination of electrostatics for αSyn fibrils

We calculated the electrostatics for PD- and MSA-PMCA fibrils using the APBS/PDB2PQR server (https://server.poissonboltzmann.org/)^68,69^. Therefore, we used our atomic models and assembled 60 peptides into a fibril, imposing the helical symmetry reported in Table 1. For interpretation of the results, we focused on the central slices of the fibrils. APBS calculations for polymorphs 2A and 2B^20^ were conducted analogously.

### Statistics and reproducibility

The current study is based on work published previously^6^ in which αSyn aggregates were amplified by PMCA from five PD and five MSA patients and extensively characterized by CD, EM, EPR, fluorescence dyes and HD-exchange coupled to NMR spectroscopy. This previous study identified aSyn aggregates amplified from PD patient #1 and MSA patient #1 as most representative of the structural properties of aSyn in aggregates amplified from the different PD and MSA patient brains, respectively. aSyn aggregates amplified by PMCA from PD patient #1 and MSA patient #1 were therefore selected and investigated in cells and structurally characterized by cryoEM.

For cryo-EM structure determination, one data set was collected for each of the MSA and PD samples. For the cell assay (Fig. 1), the aggregates added to the cells were taken from the very same sample that was used for cryoEM. Protein levels in mouse primary oligodendrocytes were measured in three independent experiments with duplicate samples/conditions within each experiment. Figure 1c shows mean and standard error (SE) of the protein levels, with significance levels *p < 0.05; **p < 0.01 obtained by Student’s unpaired *t*-test.

## Supplementary information

Supplemental Information

Description of Additional Supplementary Files

Supplementary Data 1

## Data Availability

Atomic models for the PD-PMCA and MSA-PMCA αSyn fibrils have been deposited to the Protein Data Bank (PDB) under accession codes 7OZG and 7OZH, respectively. Furthermore, corresponding reconstructed density maps have been deposited at the EMDataBank under acccesion codes 13123 and 13124, respectively. The source data used to generate Fig. 1c is included in Supplementary Data 1. All other data are available from the corresponding authors on reasonable request
