# Supplementary material for: Quaternary structure of patient-homogenate amplified α-synuclein fibrils modulates seeding of endogenous α-synuclein
Source: Commun Biol. 2022 Sep 30;5:1040. doi: 10.1038/s42003-022-03948-y (PMC9525671; doi:10.1038/s42003-022-03948-y)
Supplement: Supplementary file 1 — Supplemental Information [file 42003_2022_3948_MOESM1_ESM.pdf]

Supplemental Information

Quaternary structure of patient-homogenate amplified α-synuclein fibrils modulates seeding of endogenous α-synuclein

Benedikt Frieg[1#], James A. Geraets[1#], Timo Strohäker[2], Christian Dienemann[3], Panagiota Mavroeidi[4], Byung Chul Jung[5,+], Woojin S. Kim[6,7], Seung-Jae Lee[5], Maria Xilouri[4], Markus Zweckstetter[2,8,*], and Gunnar F. Schröder[1,9,*]

[1] Institute of Biological Information Processing (IBI-7: Structural Biochemistry), Forschungszentrum Jülich GmbH, Jülich, Germany.

[2] German Center for Neurodegenerative Diseases (DZNE), Göttingen, Germany.

[3] Department of Molecular Biology, Max Planck Institute for Multidisciplinary Sciences, Göttingen, Germany.

[4] Center of Clinical, Experimental Surgery, & Translational Research, Biomedical Research Foundation of the Academy of Athens (BRFAA) 4, Soranou Efesiou Street, Athens 11527, Greece.

[5] Department of Biomedical Sciences, Neuroscience Research Institute, College of Medicine, Seoul National University, Seoul 03080, Korea.

[6] Brain and Mind Centre and School of Medical Sciences, Faculty of Medicine and Health, The University of Sydney, Sydney, NSW, Australia.

[7] School of Medical Sciences, University of New South Wales & Neuroscience Research Australia, Randwick NSW 2031, Australia.

[8] Department for NMR-based Structural Biology, Max Planck Institute for Multidisciplinary Sciences, Göttingen, Germany.

[9] Physics Department, Heinrich Heine University Düsseldorf, Düsseldorf, Germany.

[+]present address: Nutritional Sciences and Toxicology Department, University of California Berkeley, Berkeley, CA 94720

Supplemental Figures

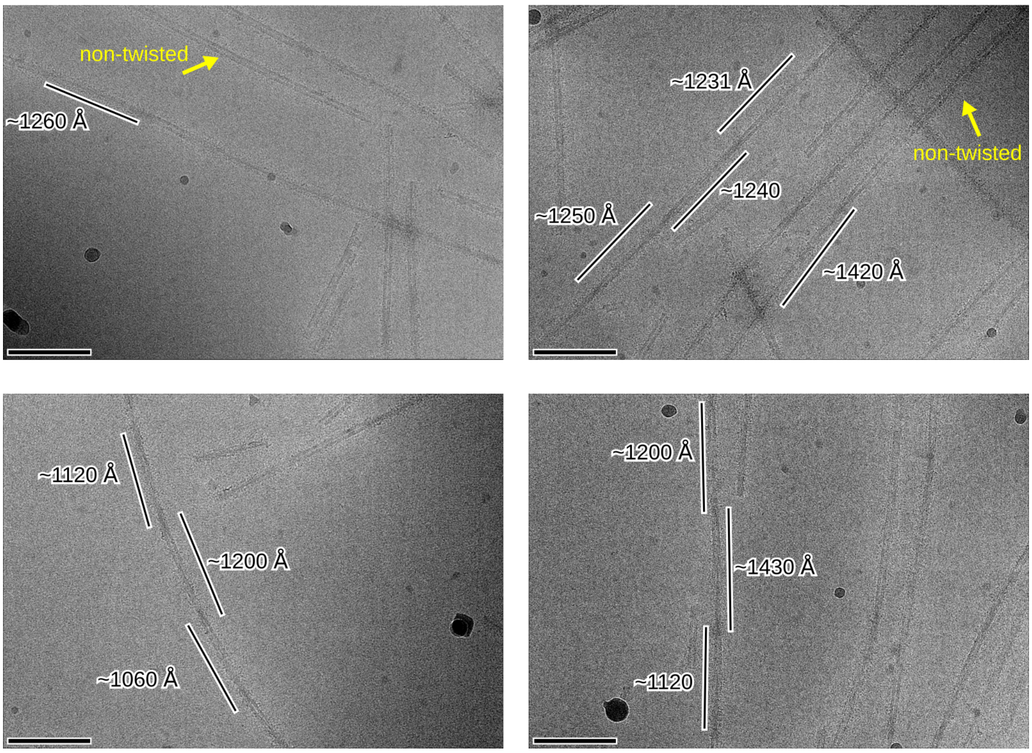

Figure S1: Representative micrographs with MSA-type αSyn fibrils.
Micrographs showing twisted (labeled with cross over distances) and non-twisted αSyn fibrils amplified from MSA patient's material. Scale bars, 100 nm.

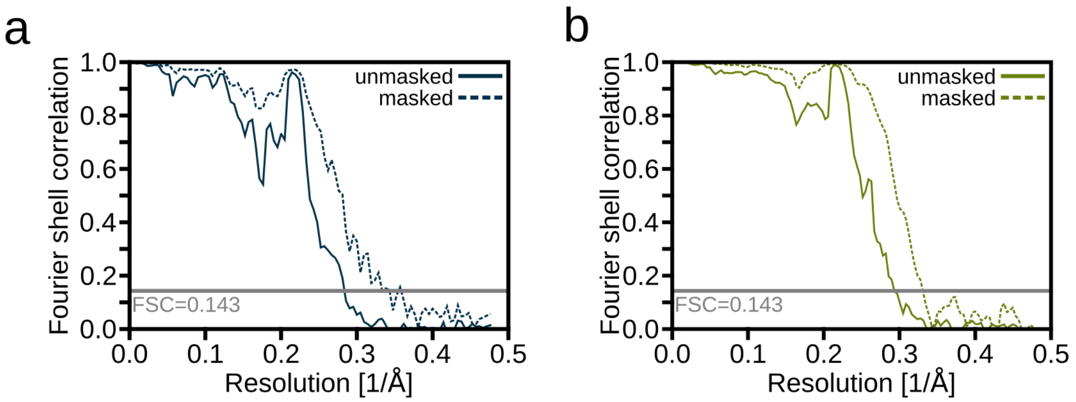

Figure S2: Fourier shell correlation curves.

The Fourier shell correlation curves are shown between two independently refined unmasked (solid lines) and masked (dashed lines) half-maps. The *z*-percentage was 0.3 in the case of PD (**a**) and 0.1 in the case of in MSA (**b**), respectively.

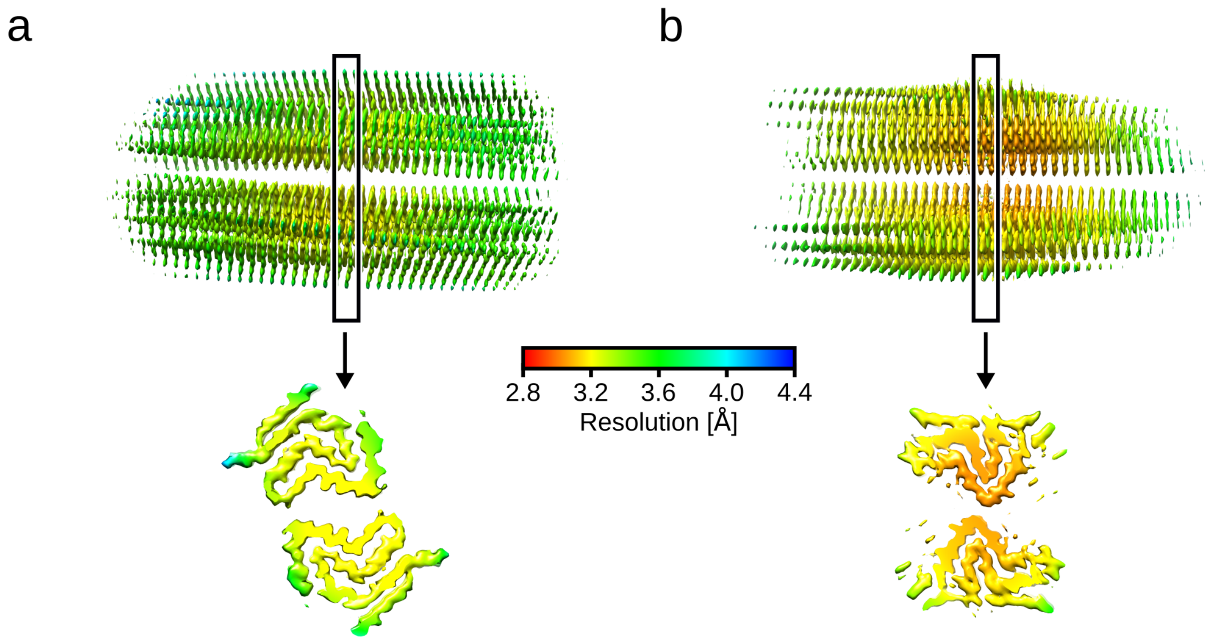

Figure S3: Local resolution estimation.
Reconstructed map of PD (**a**) and MSA (**b**) fibrils colored according to the local resolution estimation (see color scale). The lower panel shows a cross-section of the central region.

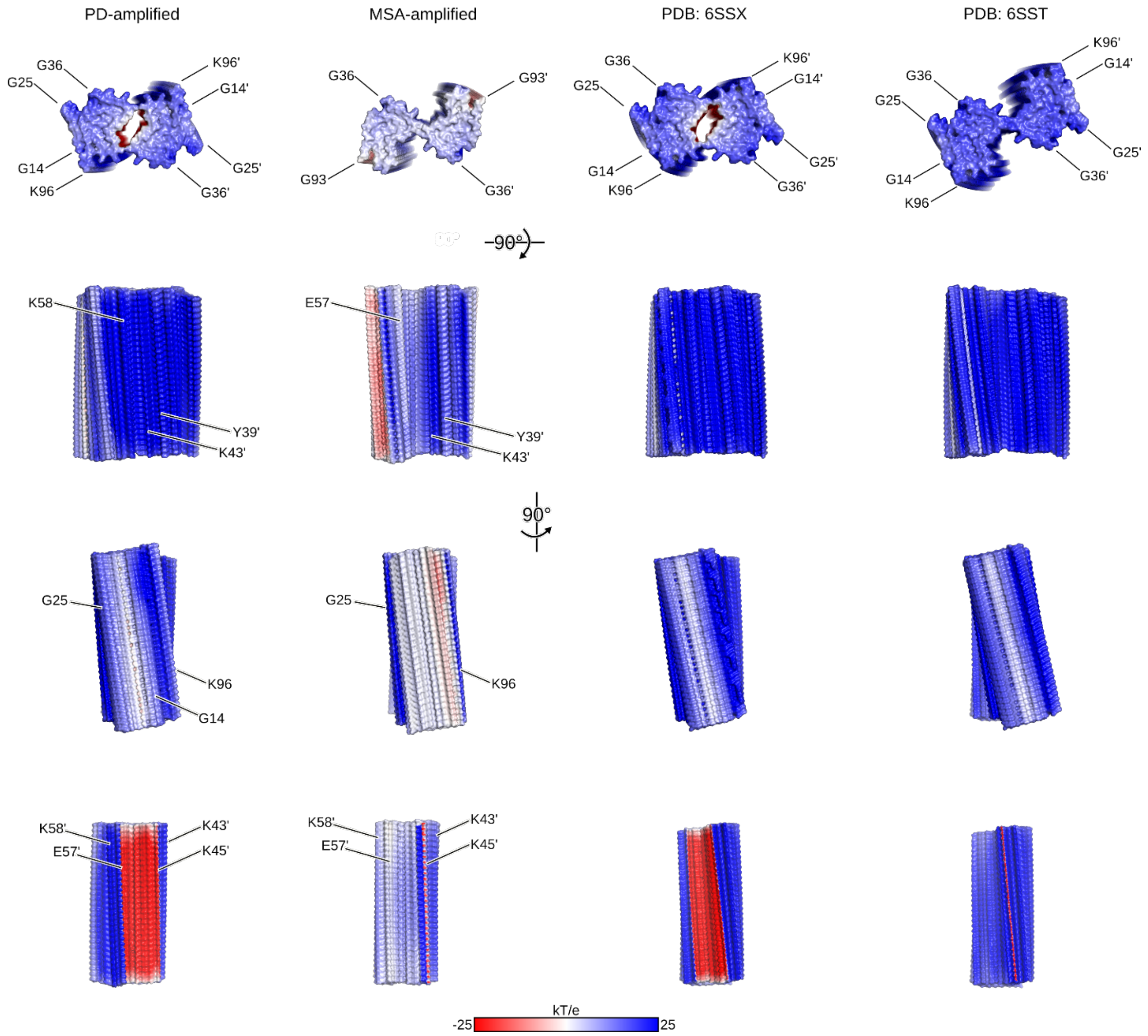

Figure S4: Electrostatic surface potential.

The cryo-EM structures of amplified αSyn fibrils from PD (left) and MSA (right) brain and two αSyn fibrils of in vitro aggregated recombinant protein (Guerrero-Ferreira et al., 2019) viewed from different angles. The calculated electrostatic potential was mapped onto the surface and colored according to the color scale at the bottom. The bottom panel shows a cross section along the Z-axis, with the inter-protofilament interfaces now oriented towards the viewer. For illustrative purposes, some amino acids are explicitly labeled, with amino acids from subunit B marked with an additional prime.

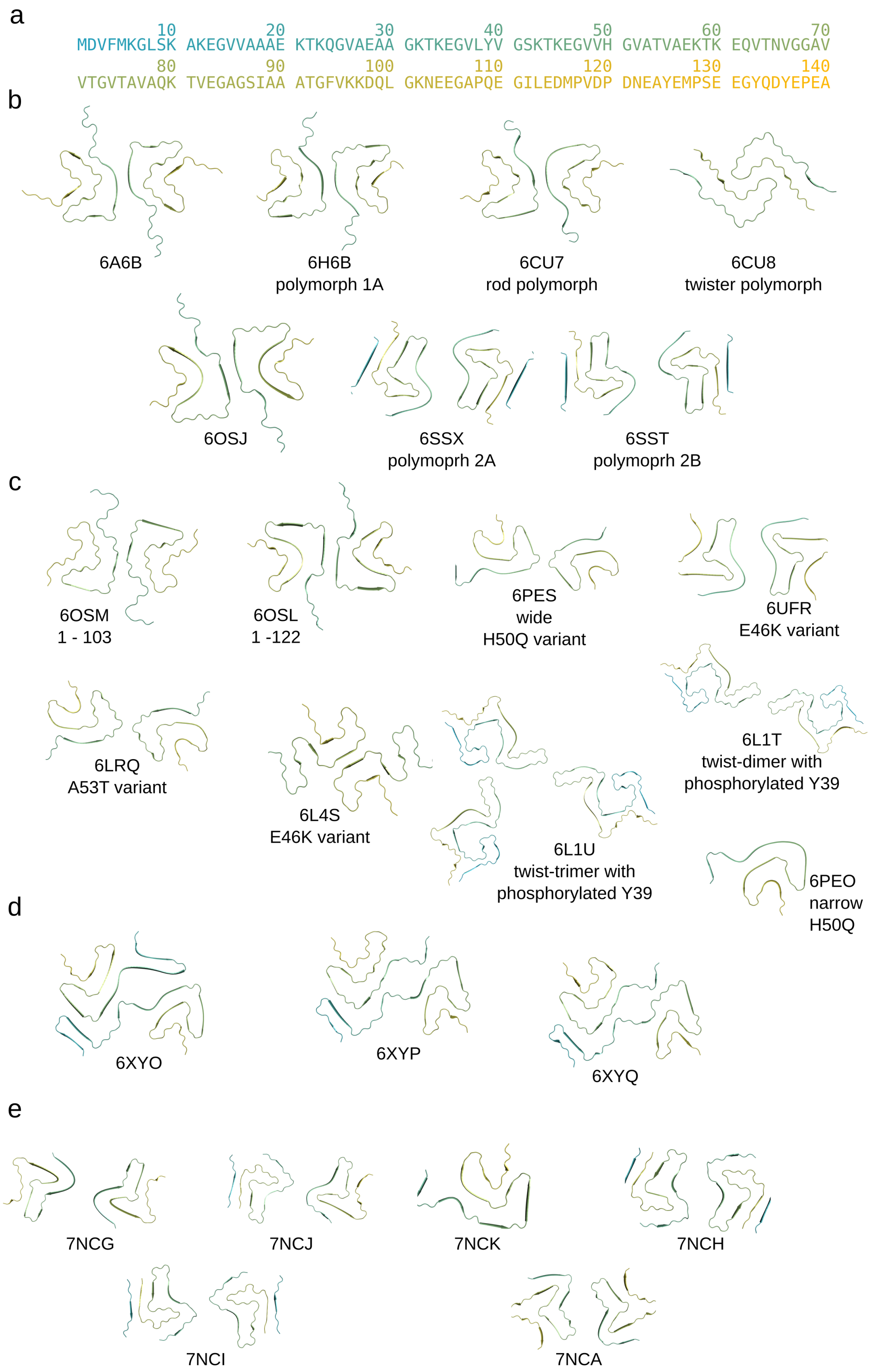

Figure S5: Summary of previously resolved αSyn structures.
a: Full-length mino acid sequence of human αSyn (UniProt: P37840). The sequence is colored from the N- to the C-terminus according to the blue-green-yellow pallet. **b-e:** Top view onto the previously resolved αSyn structures. The structures are colored according to the color code in **a**. The four-letter PDB-ID is reported with the structures. The structures can be organized into four groups, in which the fibrils are formed by recombinant full-length wild type αSyn (**b**), recombinant, truncated, or modified αSyn (**c**), αSyn extracted from brain tissue of MSA diagnosed patients (**d**), and αSyn amplified from seeded brain extracts (**e**) (for details, please see **Table S3**)

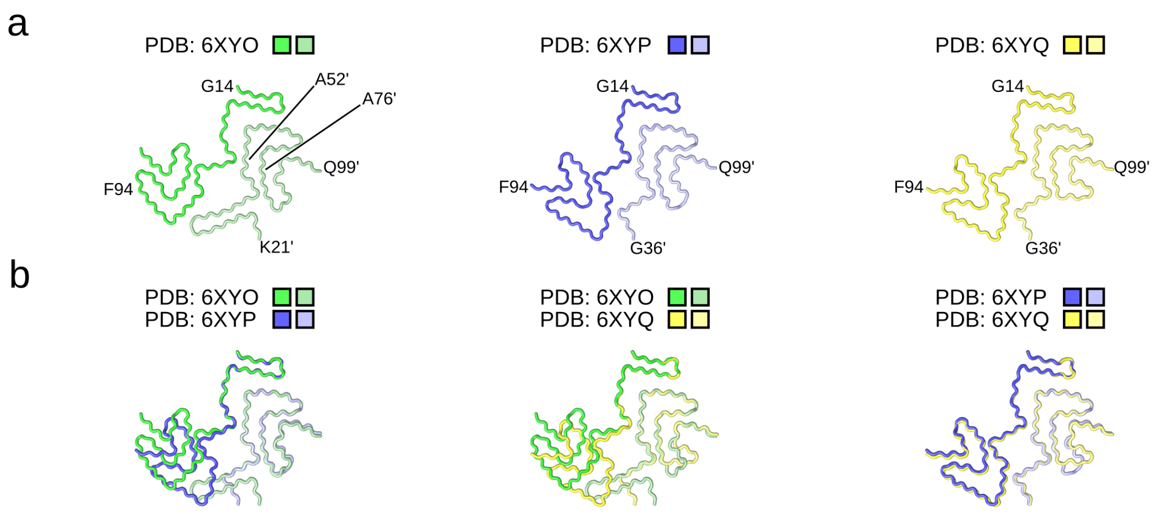

Figure S6: Comparison between *ex vivo* MSA type αSyn fibrils.
a: Top view onto the αSyn structures from the brains of individuals with MSA [1]. The protofilaments are colored in different shades of green, blue, and yellow, with amino acids from subunit B labeled with a prime. **b**: Superposition of αSyn structures from a**.** All structures were superimposed on the region extending from A52' to A57'.

Supplemental Tables

Table S1. PD-amplified αSyn fibril structure compared to the *in vitro* polymorph 2A (PDB ID 6SSX [2]) and MSA-amplified fibril structure from Lovestam et al. (PDB ID 7NCH [3]).

	**PD-amplified αSyn**	**PDB 6SSX**	**PDB 7NCH**
Overlay onto PD-amplified αSyn[a]	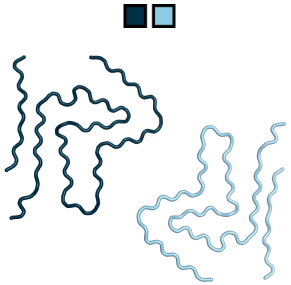	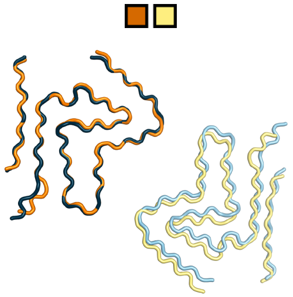	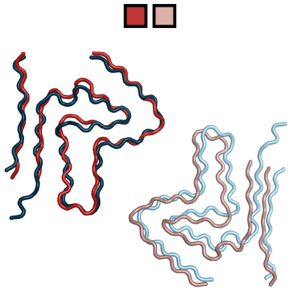
Cα RMSD to PD type αSyn [Å] (one/two protofilament)[b]	-- / --	1.03 / 1.33	1.65 / 2.65
Symmetry	C2	C2	C2
Rise [Å] / Twist [°]	4.68 / -0.78	4.80 / -0.80	4.78 / -0.86

[a] To visualize the displacement between two opposite protofilaments, only the Cα atoms of one protofilament (shown in darker colors) were aligned.

[b] Only amino acids present in both structures (mobile and reference) were considered for RMSD calculations.

Table S2. MSA-amplified αSyn fibril structure compared to the *in vitro* polymorph 2B (PDB ID 6SST [2]) and MSA-amplified fibril structure from Lovestam et al. (PDB IDs 7NCI and 7NCG [3]).

	**MSA type αSyn**	**PDB 6SST**	**PDB 7NCI**	**PDB 7NCG**
Overlay onto MSA type αSyn[a]	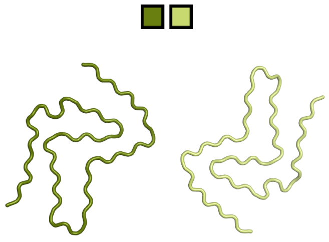	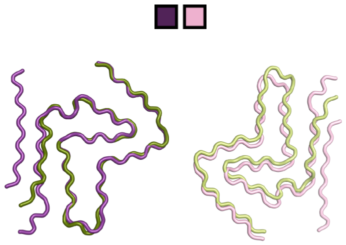	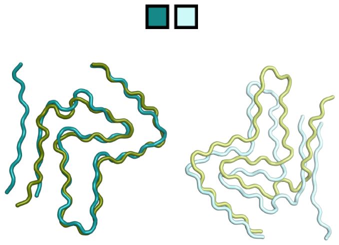	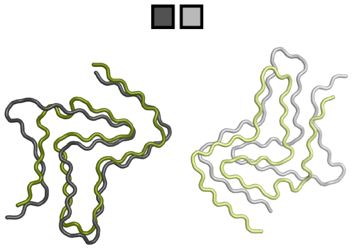
Cα RMSD to MSA type αSyn [Å] (one/two protofilament)[b]	-- / --	1.48 / 1.74	1.50 / 12.55	4.57 / 9.23
Symmetry	C1	C1	C2	C2
Rise [Å] / Twist [°]	2.37 / 179.66	2.40 / 179.55	4.75 / -0.77	4.75 / -0.95

[a] To visualize the displacement between two opposite protofilaments, only the Cα atoms of one protofilament (shown in darker colors) were aligned.

[b] Only amino acids present in both structures (mobile and reference) were considered for RMSD calculations.

Table S3: Summary of αSyn structures.

PDB-ID	Polymorph name	Variation	Ref.
**Recombinant full-length human αSyn**			
6FLT (see 6H6B)		full-length human αSyn	
6A6B		full-length human αSyn,	[4]
6H6B	polymorph 1A	full-length human αSyn	[5]
6CU8	Twister/ polymorph 1B	full-length human αSyn	[6]
6CU7	Rod	full-length human αSyn	[6]
6RTB (see 6SST)	polymorph 2B	full-length human αSyn	
6RT0 (see 6SSX)	polymorph 2A	full-length human αSyn	
6OSJ		full-length human αSyn	[7]
6SSX	polymorph 2A	full-length human αSyn	[2]
6SST	polymorph 2B	full-length human αSyn	[2]
**Recombinant truncated or modified αSyn**			
6OSM		Recombinant 1 -103 human αSyn	[7]
6OSL		Recombinant 1 - 122 human αSyn	[7]
6PES	wide	Recombinant full-length H50Q human αSyn	[8]
6PEO	narrow	Recombinant full-length H50Q human αSyn	[8]
6UFR		Recombinant full-length E46K human αSyn	[9]
6LRQ		Recombinant full-length A53T human αSyn	[10]
6L4S		Recombinant full-length E46K human αSyn	[11]
6L1T	twist-dimer fibril	Recombinant full-length phosphorylated Y39 human αSyn	[12]
6L1U	twist-trimer fibril	Recombinant full-length phosphorylated Y39 human αSyn	[12]
**αSyn extracted from brain tissue of MSA diagnosed patients**			
6XYO	MSA type II-1	MSA brain extracts, *ex vivo*	[1]
6XYP	MSA type I	MSA brain extracts, *ex vivo*	[1]
6XYQ	MSA type II-2	MSA brain extracts, *ex vivo*	[1]

αSyn amplified from seeded brain extracts

7NCG	Type 2A	Full-length alpha-synuclein fibril seeded *in vitro* by fibrils purified from MSA brain	[3]
7NCJ	Type 2AB	Full-length alpha-synuclein fibril seeded *in vitro* by fibrils purified from MSA brain	[3]
7NCK	Type 3	Full-length alpha-synuclein fibril seeded *in vitro* by fibrils purified from MSA brain	[3]
7NCH	Type 1B	Full-length alpha-synuclein fibril seeded *in vitro* by fibrils purified from MSA brain	[3]
7NCI	Type 2B	Full-length alpha-synuclein fibril seeded *in vitro* by fibrils purified from MSA brain	[3]
7NCA	Type 1A	Full-length alpha-synuclein fibril seeded *in vitro* by fibrils purified from MSA brain	[3]

Supplemental References

1. Schweighauser, M. et al. Structures of alpha-synuclein filaments from multiple system atrophy. *Nature* (2020).

2. Guerrero-Ferreira, R. et al. Two new polymorphic structures of human full-length alpha-synuclein fibrils solved by cryo-electron microscopy. *Elife* **8**(2019).

3. Lövestam, S. et al. Seeded assembly in vitro does not replicate the structures of alpha-synuclein filaments from multiple system atrophy. *Febs Open Bio* **11**, 999-1013 (2021).

4. Li, Y. et al. Amyloid fibril structure of alpha-synuclein determined by cryo-electron microscopy. *Cell Res* **28**, 897-903 (2018).

5. Guerrero-Ferreira, R. et al. Cryo-EM structure of alpha-synuclein fibrils. *Elife* **7**(2018).

6. Li, B. et al. Cryo-EM of full-length alpha-synuclein reveals fibril polymorphs with a common structural kernel. *Nat. Commun.* **9**, 3609 (2018).

7. Ni, X., McGlinchey, R.P., Jiang, J. & Lee, J.C. Structural insights into alpha-synuclein fibril polymorphism: Effects of Parkinson's disease-related C-terminal truncations. *J. Mol. Biol.* **431**, 3913-3919 (2019).

8. Boyer, D.R. et al. Structures of fibrils formed by alpha-synuclein hereditary disease mutant H50Q reveal new polymorphs. *Nat. Struct. Mol. Biol.* **26**, 1044-1052 (2019).

9. Boyer, D.R. et al. The alpha-synuclein hereditary mutation E46K unlocks a more stable, pathogenic fibril structure. *Proc. Natl. Acad. Sci. U. S. A.* **117**, 3592-3602 (2020).

10. Sun, Y.P. et al. Cryo-EM structure of full-length alpha-synuclein amyloid fibril with Parkinson's disease familial A53T mutation. *Cell Res* **30**, 360-362 (2020).

11. Zhao, K. et al. Parkinson's disease associated mutation E46K of alpha-synuclein triggers the formation of a distinct fibril structure. *Nat. Commun.* **11**(2020).

12. Zhao, K. et al. Parkinson's disease-related phosphorylation at Tyr39 rearranges alpha-synuclein amyloid fibril structure revealed by cryo-EM. *Proc. Natl. Acad. Sci. U. S. A.* **117**, 20305-20315 (2020).
