# Supplementary material for: Quaternary structure of patient-homogenate amplified α-synuclein fibrils modulates seeding of endogenous α-synuclein
Source: Commun Biol. 2022 Sep 30;5:1040. doi: 10.1038/s42003-022-03948-y (PMC9525671; doi:10.1038/s42003-022-03948-y)
Supplement: Supplementary file 2 — Description of Additional Supplementary Files [file 42003_2022_3948_MOESM2_ESM.pdf]

Description of Additional Supplementary Files

File name: Supplementary Data 1
Description: The source data behind graphs in Figure 1c.
